# CoQ10 ameliorates monosodium glutamate-induced alteration in detrusor activity and responsiveness in rats via anti-inflammatory, anti-oxidant and channel inhibiting mechanisms

**DOI:** 10.1186/s12894-019-0534-9

**Published:** 2019-10-28

**Authors:** Dalia F. El Agamy, Yahya M. Naguib

**Affiliations:** Clinical Physiology Department, Faculty of Medicine, Menoufia University, Menoufia, Egypt

**Keywords:** Detrusor overactivity, Monosodium glutamate, CoQ10, Connexin43, Nerve growth factor

## Abstract

**Background:**

Competent detrusor muscles with coordinated contraction and relaxation are crucial for normal urinary bladder storage and emptying functions. Hence, detrusor instability, and subsequently bladder overactivity, may lead to undesirable outcomes including incontinence. Multiple mechanisms may underlie the pathogenesis of detrusor overactivity including inflammation and oxidative stress. Herein, we tested the possibility that CoQ10 may have a potential therapeutic role in detrusor overactivity.

**Methods:**

Forty adult male Wistar albino rats weighing 100-150 g were used in the present study. Rats were divided (10/group) into control (receiving vehicles), monosodium glutamate (MSG)-treated (receiving 5 mg/kg MSG daily for 15 consecutive days), MSG + OO-treated (receiving concomitantly 5 mg/kg MSG and olive oil for 15 consecutive days), MSG + CoQ10-treated (receiving concomitantly 5 mg/kg MSG and 100 mg/kg CoQ10 daily for 15 consecutive days) groups.

**Results:**

MSG resulted in significant increase in bladder weight and sensitised the bladder smooth muscles to acetylcholine. MSG has also resulted in significant increase in bladder TNF-α, IL-6, malondialdehyde, nerve growth factor and connexion 43, with significant decrease in the antioxidant enzymes superoxide dismutase and catalase. Olive oil had no effect on MSG induced alterations of different parameters. Treatment with CoQ10 has resulted in a significant restoration of all the altered parameters.

**Conclusion:**

Taken together, our results suggest that CoQ10 antagonizes the deleterious effects of MSG on detrusor activity. We propose that CoQ10 could be a therapeutic strategy targeting urinary bladder dysfunction.

## Background

Normal lower urinary tract storage and voiding functions rely on a competent urinary bladder. Disturbance of the urinary bladder storage competence may result in embarrassing symptoms such as urgency, frequency, nocturia and even incontinence [1]. Most patients with symptoms such as urgency urge incontinence, frequency or nocturia are considered to be treated from overactive detrusor even without a definitive diagnosis. This was rationale based on the reluctance to subject patients to invasive urodynamic studies unless it is absolutely necessary [2]. Detrusor smooth muscle (DSM) has spontaneous action potentials and spontaneous phasic activity. There is poor electrical coupling between DSM fibers that facilitates muscle bundles to adjust their length to achieve minimum surface area/volume ratio during filling phase with no contraction or increase in intravesical pressure. A disturbance in this mechanism may give rise to synchronous activation of muscle bundles resulting in instability of the urinary bladder [3]. Several molecular mechanisms affecting bladder smooth muscle, urothelium, and nerves has been proposed to explain the complex pathophysiology. Those included altered channel activities and enhanced bladder response to chemical and mechanical stimuli [4].

Monosodium Glutamate (MSG), the sodium salt of glutamate, is one of the world’s most extensively used food additive [5]. Although MSG is considered as a bladder irritant, few data are available on the effect of MSG on the renal system and even fewer is known about the effects of MSG on the urinary bladder. MSG was found to be associated with urolithiasis and urinary tract obstruction in experimental setting [6]. MSG was capable to induce bladder epithelial hyperplasia in rats [7]. MSG increases the level of glutamate; a major excitatory neurotransmitter that plays an important role in the reflex pathways controlling the lower urinary tract functions [8]. Tonic activation of glutamate receptors contributes to the bladder overactivity [9].

Connexins (Cxs) are a family of specific proteins with plethora of functions. They may act as intercellular ion channels (gap junctions, GJs), second messengers, small signalling molecules, and conductors for electrical signals [10]. Connexins play an important role in the coordinated contraction and relaxation responses required for bladder emptying and filling [11]. Gap junction proteins are dynamic membrane proteins that have short half-lives of only few hours. The mechanisms regulating connexins turnover are complex. Various protein kinases phosphorylate connexins and phosphorylation may trigger its internalization and degradation [12]. There is a close relationship between elevated detrusor muscle Cx43 expression and detrusor muscle overactivity [13].

Coenzyme Q10, CoQ10 or ubiquinone-10, is an endogenous compound that acts as a powerful antioxidant, a crucial cofactor in the mitochondrial electron transport system, as well as a modulator of gene expression [14, 15]. CoQ10 tends to decrease both pro-inflammatory and oxidative stress markers in experimental animals [16, 17]. Interestingly, CoQ10 was shown to regulate adenosine monophosphate-activated protein kinase (AMPK) phosphorylation in a dose and time dependent manners [18, 19]. (AMPK) is an intracellular energy sensor which is activated under low cellular energy status. AMPK maintains cellular energy homeostasis and has regulatory effects on the cellular metabolism [20]. AMPK has anti-inflammatory, anti-oxidative and channel-inhibiting properties. It counteracts the biological actions of several inflammatory mediators and growth factors including those implicated in the up-regulation of Cx43 expression in the detrusor muscle and bladder overactivity. In addition, AMPK was reported to suppress Cx43 in a mouse model of cystitis [21].

Herein, we hypothesised that CoQ10 could ameliorate the deleterious effects of MSG on detrusor muscle activity. We also tested the possibility that the anti-inflammatory, antioxidant and channel inhibiting properties of CoQ10 may underlie its actions.

## Methods

### Animals and

Forty male Wistar rats weighing approximately 100–150 g were obtained from a local animal providing facility. Rats were kept under controlled temperature, humidity, and 12 h light/dark cycles. Rats were given access to standard rodent chow and water ad libitum. To allow proper acclimatization, rats were kept for 10 days prior to experimentation. Animal care and use were approved by the Ethics Committee of the Faculty of Medicine-Menoufia University-Egypt.

### Experimental design

All experiments were carried in accordance with the Guide for the Care and Use of Laboratory Animals published by the US National Institutes of Health (NIH Publication no. 85–23, revised in 1996). Rats were divided randomly (10 rats/group) following the acclimatization period into: (1) control group: rats received 0.9% saline orally via gavage, and olive oil via intra-peritoneal injection, (2) MSG-treated group: rats received single daily dose (5 g/kg body weight dissolved in 0.9% saline via oral gavage) of monosodium glutamate (MSG, Sigma-Aldrich, UK) for 15 consecutive days, (3) MSG and olive oil (OO)-treated group: rats received MSG (5 g/kg body weight dissolved in 0.9% saline via oral gavage), and OO via intra-peritoneal injection (IP) for 15 consecutive days, and (4) MSG and CoQ10-treated group: rats received MSG (5 g/kg body weight dissolved in 0.9% saline via oral gavage), and CoQ10 (Sigma-Aldrich, UK, 10 mg/kg body weight dissolved in olive oil IP) daily for 15 consecutive days.

### Blood sampling

Rats were fasted overnight after the last doses of MSG and CoQ10. Rats were then anaesthetised by injecting sodium thiopental (STP) (60 mg/kg IP). Blood samples were collected via cardiac puncture, left to clot for 10–15 min and then centrifuged at 4000 rpm for another 10 min. Serum samples were stored at − 20 °C for subsequent analysis of serum tumor necrosis factor alpha (TNF-α) and interleukin 6 (IL-6). All rats were then scarified by cervical dislocation.

### Dissection of urinary bladder

Instantaneously after the rats were sacrificed the urinary bladder was dissected, weighed, and transferred to Kreb’s solution. The bladder was incised longitudinally from the base to the dome. Then the bladder was opened up to form a flat sheet and the base and the top of the dome were cautiously excised. The sheet was then cut longitudinally into three equal parts; for tissue homogenization, Cx43 RT-PCR, and for the assessment of the in vitro bladder contractile activity.

### Preparation of urinary bladder tissue homogenate

Bladder tissue was homogenized in ice cold phosphate buffer (pH 7.4). Using a surgical scalpel, bladder tissue was cross-chopped into fine slices, suspended in chilled 0.25 M sucrose solution and rapidly blotted on a filter paper. Mincing and homogenization of tissue were performed to release soluble proteins in ice-cold Tris hydrochloride buffer (10 mM, pH 7.4). Tissue homogenate was centrifuged at 7000 rpm for 20 min and the supernatant was collected and stored at − 20 °C for subsequent estimation of malondialdehyde (MDA), superoxide dismutase (SOD), catalase (CAT), and nerve growth factor (NGF).

### Assessment of the contractile activity of urinary bladder strips

One third of the urinary bladder was cut into 3–4 longitudinal strips measuring 2–4 * 6–12 mm depending on its size. The strips were transferred into a Petri-dish containing Krebs’ solution aerated with carbogen (95% oxygen and 5% carbon dioxide) at room temperature. Each strip was then suspended in a 10 ml organ bath containing the freshly prepared Krebs’ solution, maintained at 37 °C and continuously bubbled with carbogen. The preparation was attached to a force transducer (Grass, USA), and isometric tension was recorded by physiograph (MKIII-S Universal Coupler, Narco Bio-System, USA). The strip was allowed to equilibrate for 60 min. A resting tension of 1 g was maintained throughout the experiment. Following equilibration, phasic activity of the tissues was recorded and the response of the tissue to 10^− 5^ M acetylcholine (cholinergic receptor agonist) was recorded [22].

### Biochemical assays

Serum levels of TNF-α and IL-6 (Quantikine® ELISA, R&D Systems Inc., MN, USA), and tissue homogenate level of NGF (MyBioSource, Inc., USA) were measured by enzyme linked immune sorbent assay (ELISA) technique according to the manufacturer’s instructions. Measurement was performed with ELISA automatic optical reader and the absorbance was taken at 450 nm (SUNRISE Touchscreen, TECHAN, Salzburg, Austria). Tissue level of MDA (QuantiChrom™, BioAssay Systems, USA), CAT (EnzyChrom™, BioAssay Systems, USA), and SOD (EnzyChrom™, BioAssay Systems, USA) were determined by colorimetric method on a Jenway Genova autoanalyser (UK).

### Quantification of Cx43 mRNA

Total RNA was extracted from rat bladders using TRI reagent (Sigma-Aldrich, UK). Extracted RNAs were reverse transcribed using the high capacity RNA-to-cDNA kit (Applied Biosystems, Foster City, CA, USA) according to the manufacturer’s instructions. Real-time RT-PCR was performed using a Biosystem 7300 (Applied Biosystems, CA, USA). To quantify changes in gene expression, the comparative Ct method was used to calculate the relative-fold changes normalized relative to the housekeeping gene GAPDH. The gene specific primers for Cx43 were: sense TGGGGGAAAGGCGTGAG and antisense CTGCTGGCTCTGCTGGAAGGT, while primers for GAPDH were: sense TGAAGGTCGGTGTGAACGGATTTGGC, and antisense CATGTAGGCCATGAGGTCCACCAC. Results are shown as the mean of three samples, with each sample assayed in duplicate.

### Statistical analysis

Results are expressed as mean ± standard deviation (SD). Kolmogorov-Smirnov test was performed on all data sets to ensure normal distribution (*p* > 0.5). Student t-test or repeated-measures Analysis of Variances (ANOVA) were used for statistical analysis of the different groups whichever appropriate, using Origin® software and the probability of chance (*p* values). *P* values < 0.05 were considered significant.

## Results

### Urinary bladder weight and detrusor response to acetylcholine

**Fig. 1:**
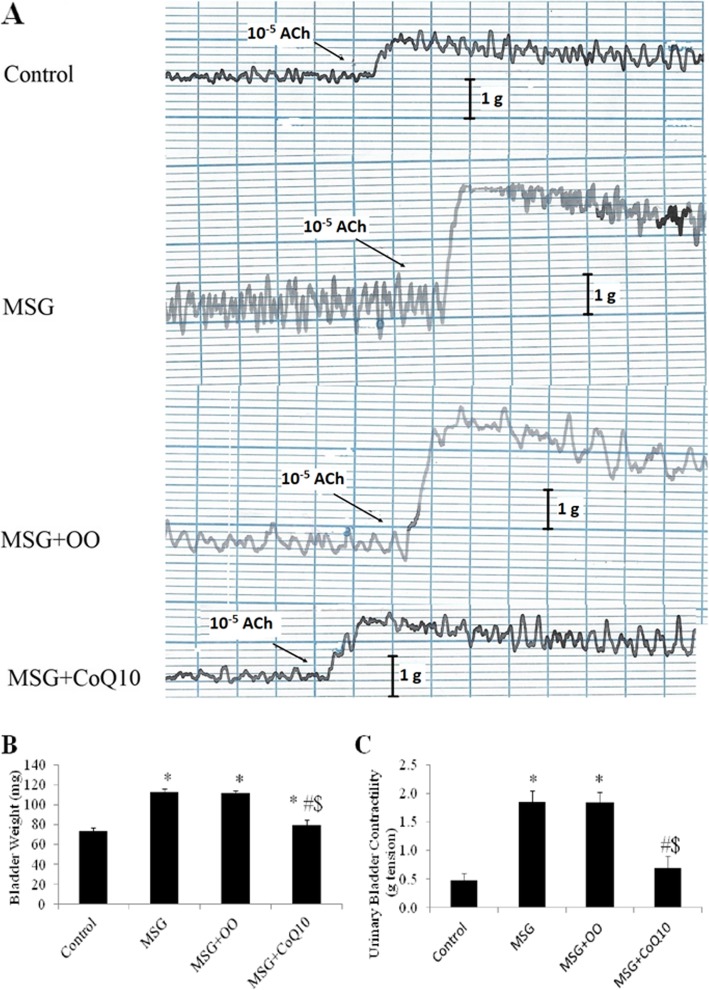
CoQ10 restores detrusor smooth muscle contractile response and bladder weight in MSG treated rats. **a** Representative traces of detrusor muscle response to acetylcholine (Ach) in monosodium glutamate (MSG), MSG + OO, and MSG + CoQ10 treated rats. **b** Effect of CoQ10 treatment on urinary bladder strips contractility in control, monosodium glutamate (MSG), MSG + OO, and MSG + CoQ10 treated rats. **c** Effect of CoQ10 treatment on urinary bladder weight in control, monosodium glutamate (MSG), MSG + OO, and MSG + CoQ10 treated rats. * Significant when compared to control group. ^#^ Significant when compared to MSG group. ^$^ Significant when compared to MSG + OO group (*n* = 10)

Treatment with MSG resulted in significant increase in the weight of the urinary bladder when compared to the control group (113 ± 3.2 vs 73.9 ± 2.9 mg, *P* < 0.001). Insignificant difference was observed in bladder weight in the MSG + OO group (111.9 ± 2.8 mg) when compared to the corresponding value in the MSG treated group (*P* = 0.926), while urinary bladder weight was significantly higher (*P* < 0.001) if compared to the control group. The weight of the urinary bladder was significantly decreased in the MSG + CoQ10 group (79.8 ± 5.2 mg) when compared to the MSG or MSG + OO treated groups (*P* < 0.001), albeit it was still significantly higher when compared to the control group (*P* = 0.016). Detrusor smooth muscle contractility in response to acetylcholine (ACh) was significantly increased in MSG treated group when compared to the control group (1.9 ± 0.19 vs 0.5 ± 0.11 mg tension, *P* < 0.001). Detrusor muscle responsiveness was insignificantly changed in MSG + OO (1.8 ± 0.19 mg tension) when compared to the MSG treated group (*P* = 0.992), and significantly increased if compared to the control group (*P* < 0.001). Detrusor responsiveness was significantly decreased in the MSG + CoQ10 treated group (0.7 ± 0.21 mg tension) when compared to MSG or MSG + OO treated groups (*P* < 0.001). There was insignificant difference between the MSG + CoQ10 treated group when compared to the control group (*P* = 0.144) (Fig. 1).

### Serum level of TNF-α and IL-6

**Fig. 2:**
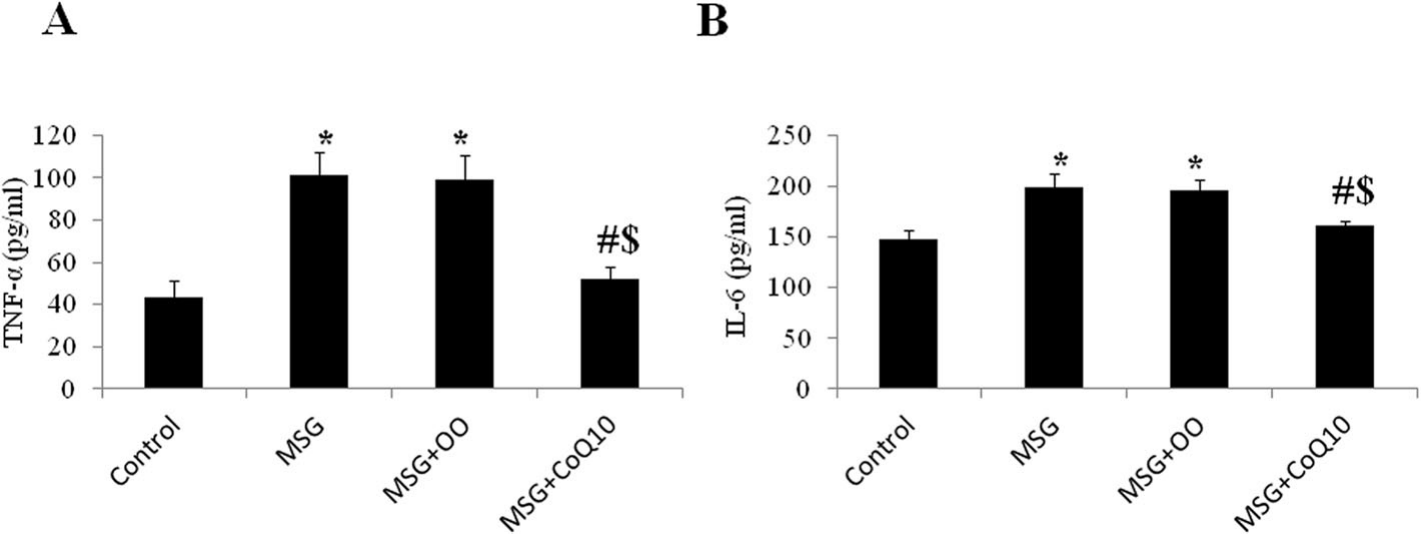
CoQ10 attenuates MSG-induce inflammation. **a** Effect of CoQ10 treatment on serum level of TNF-α in control, monosodium glutamate (MSG), MSG + OO, and MSG + CoQ10 treated rats. **b** Effect of CoQ10 treatment on serum level of IL-6 in control, monosodium glutamate (MSG), MSG + OO, and MSG + CoQ10 treated rats. * Significant when compared to control group. ^#^ Significant when compared to MSG group. ^$^ Significant when compared to MSG + OO group (*n* = 10)

MSG treatment resulted in significant increase in serum level of TNF-α and IL-6 (101.8 ± 10.9 pg/ml and 199.5 ± 12.6 pg/ml respectively), when compared to the control group (43.5 ± 7.9 and 148.5 ± 8.78 respectively, *P* < 0.001). In the MSG + OO treated group, there was insignificant difference in TNF-α and IL-6 levels (99 ± 11.9 pg/ml and 195.9 ± 10.5 pg/ml respectively) when compared to the MSG treated group (*P* = 0.938 and *P* = 0.873 respectively), while they were significantly higher when compared to the control group (*P* < 0.001). The MSG + CoQ10 treated group had significantly lower levels of TNF-α and IL-6 (51.9 ± 5.9 pg/ml and 161.4 ± 4.4 pg/ml respectively) when compared to the MSG or MSG + OO treated groups (*P* < 0.001), while there was insignificant difference if compared to the control group (*P* = 0.313 and *P* = 0.054 respectively) (Fig. 2).

### Urinary bladder homogenate level of MDA, SOD, and CAT activities

**Fig. 3:**
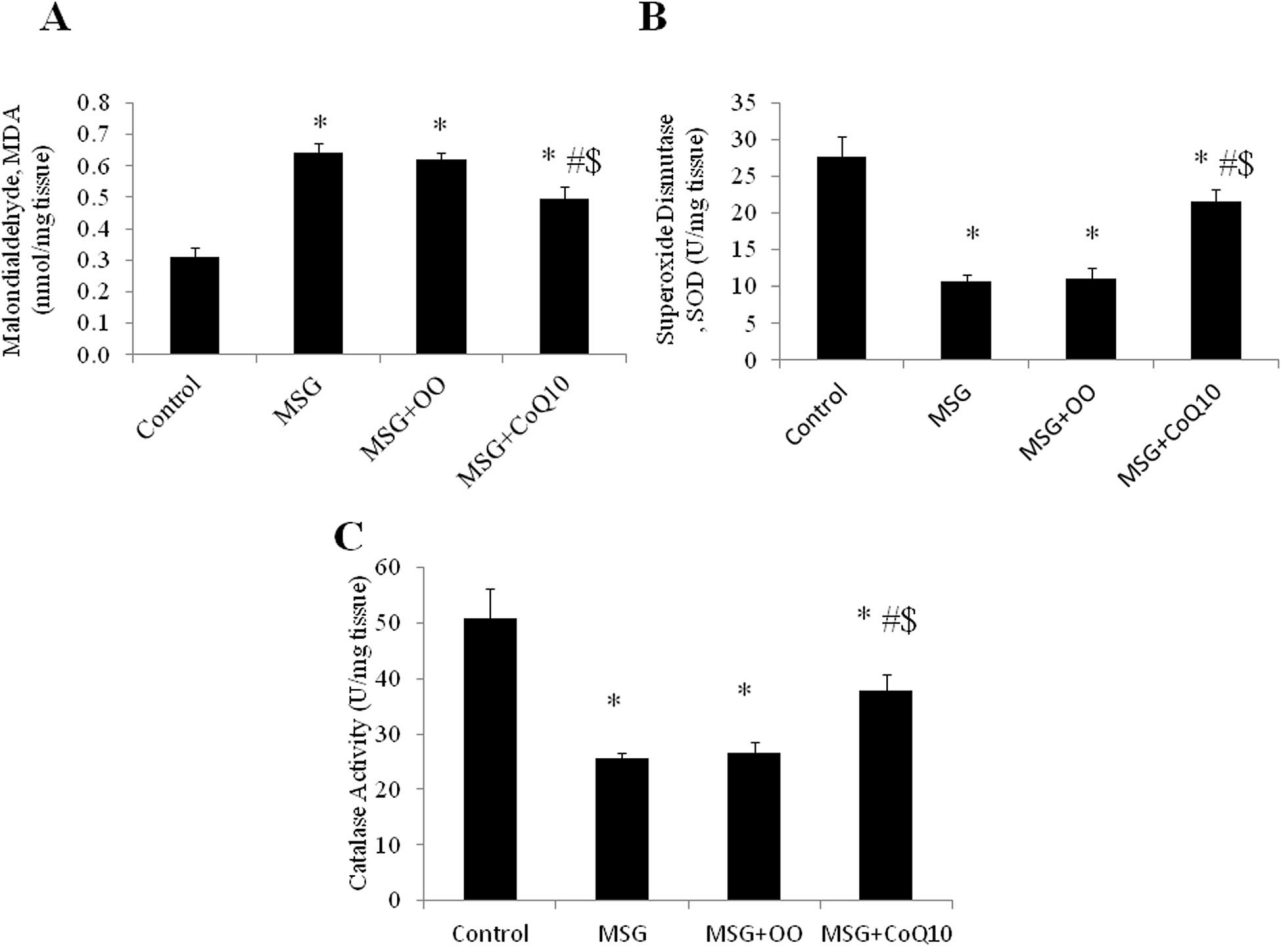
CoQ10 improves tissue oxidative status in the urinary bladder of MSG-treated rats. **a** Effect of CoQ10 treatment on tissue level of MDA in control, monosodium glutamate (MSG), MSG + OO, and MSG + CoQ10 treated rats. **b** Effect of CoQ10 treatment on tissue SOD activity in control, monosodium glutamate (MSG), MSG + OO, and MSG + CoQ10 treated rats. **c** Effect of CoQ10 treatment on tissue catalase activity in control, monosodium glutamate (MSG), MSG + OO, and MSG + CoQ10 treated rats. * Significant when compared to control group. ^#^ Significant when compared to MSG group. ^$^ Significant when compared to MSG + OO group (*n* = 10)

Tissue level of MDA was significantly higher in the MSG treated group when compared to the control group (0.64 ± 0.03 vs 0.31 ± 0.03 nmol/mg tissue, *P* < 0.001). The MSG + OO treated group showed insignificant difference in tissue level of MDA (0.62 ± 0.02 nmol/mg tissue) when compared to the MSG treated group (*P* = 0.6), while MDA level was significantly higher when compared to the control group (*P* < 0.001). The MSG + CoQ10 group had significantly lower level of MDA (0.49 ± 0.04 nmol/mg tissue) when compared to the MSG or MSG + OO treated groups (*P* < 0.001), while it was still significantly higher than the corresponding value in the control group (*P* < 0.001). On the contrary, the enzyme activity of tissue SOD and CAT in the MSG treated group was significantly lower than the control group (10.6 ± 1.06 and 25.6 ± 1.06 vs 27.8 ± 2.66 and 50.9 ± 5.33 U/mg tissue respectively, *P* < 0.001). In the MSG + OO group, SOD and CAT enzymes activity levels were insignificantly different (11.1 ± 1.46 and 26.5 ± 1.93 U/mg tissue respectively) when compared to the MSG treated group (*P* = 0.945 and *P* = 0.948 respectively), while enzymes activity levels were significantly lower when compared to control group (*P* < 0.001). In the MSG + CoQ10 group, SOD and CAT activity levels were significantly higher when compared to the MSG or MSG + OO treated groups (21.6 ± 1.68 and 37.8 ± 2.9 U/mg tissue respectively, *P* < 0.001). SOD and CAT levels were significantly lower in the MSG + CoQ10 group when compared to the control group (*P* < 0.001) (Fig. 3).

### Urinary bladder homogenate level of NGF

**Fig. 4:**
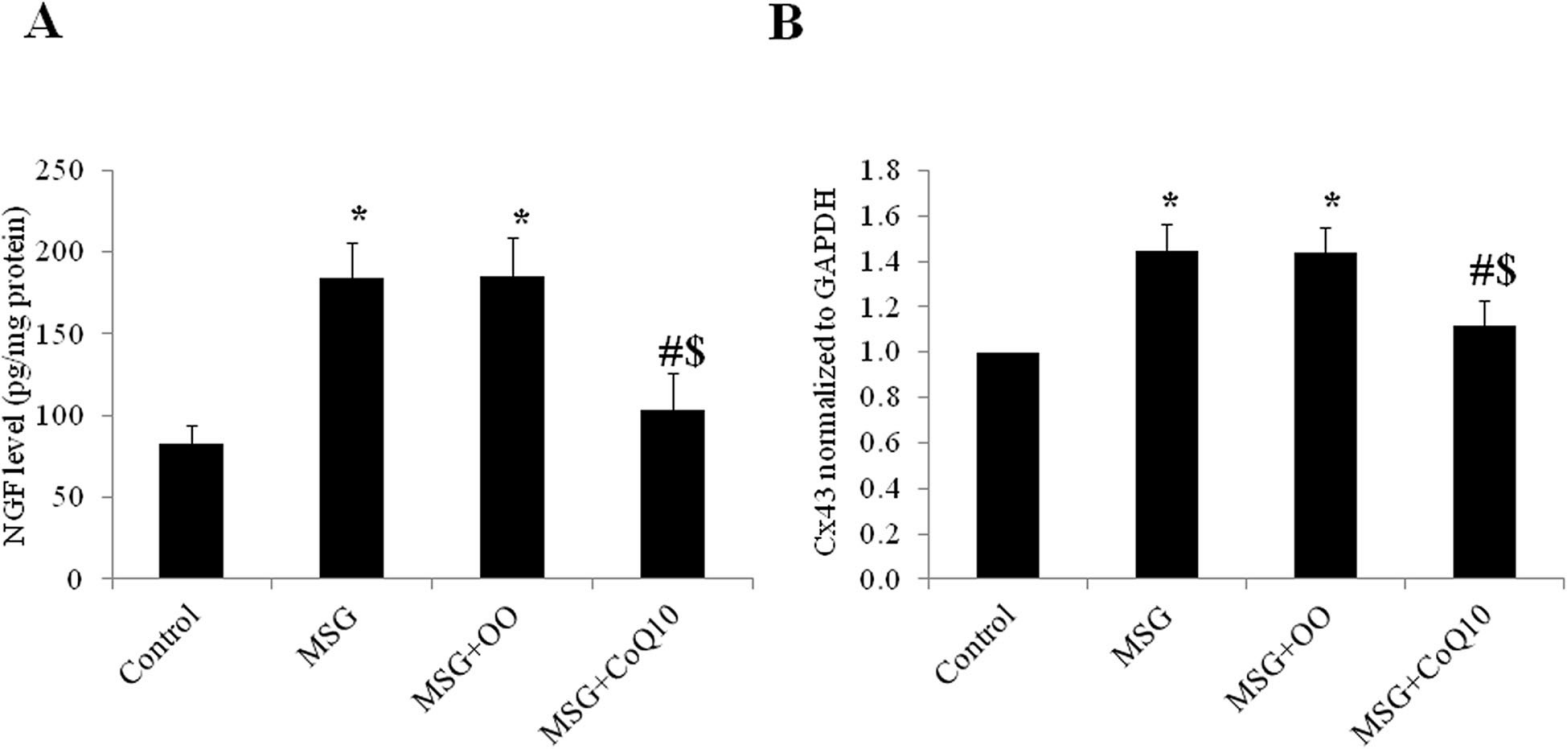
CoQ10 decreases bladder NGF tissue level and Cx43 expression. **a** Effect of CoQ10 treatment on tissue level of nerve growth factor (NGF) in control, monosodium glutamate (MSG), MSG + OO, and MSG + CoQ10 treated rats. **b** Effect of CoQ10 treatment on urinary bladder connexin43 (Cx43) expression in control, monosodium glutamate (MSG), MSG + OO, and MSG + CoQ10 treated rats. * Significant when compared to control group. ^#^ Significant when compared to MSG group. ^$^ Significant when compared to MSG + OO group (*n* = 10)

The tissue level of NGF was significantly higher in the MSG treated group when compared to the control group (184.1 ± 21.6 vs 82.3 ± 11.9 pg/mg protein, *P* < 0.001). There was insignificant difference in NGF level MSG + OO group (185.6 ± 23.06 pg/mg protein) when compared to the MSG treated group (*P* = 0.999), while NGF level was significantly higher when compared to the control group (*P* < 0.001). NGF level was significantly lower in the MSG + CoQ10 treated group (103.9 ± 22.4 pg/mg protein) when compared to MSG or MSG + OO treated groups (*P* < 0.001), while there was insignificant difference if compared to the group control (*P* = 0.167) (Fig. 4a).

### RT-PCR of Cx43 mRNA level in rat bladder

Urinary bladder Cx43 expression was significantly up-regulated in the MSG treated group when compared to the control group (1.45 ± 0.11 vs 1, *P* < 0.001). There was insignificant difference in Cx43 expression in the MSG + OO group (1.44 ± 0.11) when compared to MSG treated group (*P* = 0.992), while the expression was significantly higher when compared to control group (*P* < 0.001). Cx43 expression in the MSG + CoQ10 treated group was significantly down-regulated (1.12 ± 0.12) when compared to the MSG or MSG + OO treated groups (*P* < 0.001). There was insignificant difference in Cx43 expression between MSG + CoQ10 treated and the control group (*P* = 0.112) (Fig. 4b).

## Discussion

Monosodium glutamate is a widely used flavour enhancer and food additive that may impact several physiological functions [23]. Although it has been reported earlier that MSG may induce urinary bladder endothelial hyperplasia and altered motility [7, 24], the impact of MSG on the urinary bladder has not been properly studied. We show here that MSG may affect detrusor muscle contractility and responsiveness. We also show a potential role of the AMPK activator CoQ10 in ameliorating MSG-induced detrusor overactivity. We also show that CoQ10 exerts this action via modulating the expression of Cx43, decreasing NGF level, anti-inflammatory and anti-oxidative effects.

The present study shows that the MSG treated rats had increased urinary bladder sensitization to acetylcholine as well as increased bladder weight when compared to the control rats. Excitingly, these findings were minimized in the CoQ10 treated rats. The urinary bladder wall is lined by bundles of smooth muscle fibers; the detrusor muscle. There is poor electrical coupling between the smooth muscles bundles; a sensible property of the urinary bladder that allows the detrusor to remain quiescent with little change in intravesical pressure as the bladder fills during the urine storage phase [25]. The expression of connexins in detrusor is lower than other tissues such as the myocardium. Therefore, it does not form an effective electrical functional syncitium, allowing bladder filling with no contraction or rise of intravesical pressure. However, if the electrical activity was well coupled, intravesical spontaneous pressure changes is expected with synchronous activation of the urinary bladder and bladder instability [3]. Oxidative stress impairs the contractile responses of tissue to different agents; oxidative stress impairs the contractile response of corpus cavernosum strips and aorta rings in response to phenylephrine. The effect was abolished by the use of the anti-oxidant L-carnitine [26]. Oxidative stress was found to be a key factor in the development of detrusor overactivity in atherosclerosis-induced chronic bladder ischemia [27]. Bladder hyperactivity could be due to sensitization of afferent pathways, increased tissue damaging molecules such as NGF and prostaglandins, and up-regulation of Cx43 expression [28, 29]. Interestingly, CoQ10 has been shown to restore contractile responses to all form of stimulation in a rabbit model of obstructive bladder dysfunction [30]. Therefore, we expected that it may have a beneficial role in the restoration of normal bladder activity altered by MSG treatment. Tissue hypertrophy can be a resultant of increased inflammatory mediators, oxidative stress markers and growth factors. ROS activate a wide variety of hypertrophy signaling kinases and transcription factors [31], and is a causative factor for increased bladder weight in diabetic rats [32]. Inflammatory cytokines and growth factors have the potential to induce bladder hypertrophy and remodeling [33].

In the present study, the urinary bladder of MSG-treated rats had significantly higher levels of IL-6 and TNF-α when compared with the controls. IL-6 and TNF-α levels were significantly reduced following CoQ10 treatment. Several previous studies have shown that excess exposure to glutamate rises the level proinflammatory cytokines TNF-α and IL-6. Xu et al. reported that exogenous glutamate enhanced the inflammatory responses in brain and intestine at the transcriptional level [34]. MSG administration activates peroxisome proliferator-activated receptors that have a potential role in the control of inflammation and the impairment of pro-inflammatory cytokine signalling pathways in the liver and fat tissue [35, 36]. Inflammatory cytokines play a key role in the modulation of connexins expression and the pathogenesis of urinary bladder dysfunction [37, 38]. CoQ10 has anti-inflammatory and immunomodulatory properties. CoQ10 has been shown to reduce TNF-α, IL-2 and IL-6 levels in different settings [39]. Therefore, treatment with CoQ10 could attenuate the MSG-induced rise in proinflammatory status. Increased bladder lipid peroxidation and decreased antioxidant enzymes activity in MSG-treated rats were observed in the present study. MSG increased bladder level of MDA, and decreased tissue activity of SOD and CAT enzymes. MSG induced lipid peroxidation and increased activities of CAT and SOD in the liver of MSG-treated animals [40, 41]. CoQ10 has been shown to decrease bladder MDA level with subsequent prolongation of micturition frequency and increase of bladder capacity in a rat model of chronic bladder ischemia [42].

In the present study the level of NGF in the bladder tissue homogenate was significantly increased in the MSG-treated rats. This was counteracted by the treatment with CoQ10. NGF is a small protein that plays physiological and pathophysiological roles in the lower urinary tract. It normally exits in the afferent nerves and ganglia of the bladder wall; and either plays an important role in normal sensation of bladder distension, or sensitizes the afferent nerves to induce bladder hyperactivity [43]. Elevation of several pro-inflammatory cytokines such as TNF-α was associated with increased NGF mRNA over-expression and elevated NGF production [44, 45]. Nevertheless, previously published data demonstrated that an increase in NGF expression even without associated inflammation sensitizes the visceral reflex pathways leading to bladder overactivity [46]. Overproduction of NGF is involved in urgency and bladder dysfunction [47]. Moreover, NGF induces detrusor overactivity, modulates urothelial response to inflammation and sensory threshold of urgency, and is involved in abnormal afferent signaling and bladder sensation [48]. Cushing et al. reported that NGF increases Cx43 phosphorylation and function and regulates intercellular communication between neurons during nervous system development and repair [49]. NGF also increased Cx43 expression in various other tissues such as pulmonary arteries [50], ovaries [51], and atrial myocytes [52].

In our hands, an increase in the expression of Cx43 mRNA was evident following MSG treatment. This was countered by treatment with CoQ10. Urinary bladder Cx43 is a gap junction protein that enhances intercellular electrical and chemical transmission and increases bladder response to cholinergic stimuli. Cx43 over-expression may lead to a decrease in the functional bladder capacity resulting in an increase in frequency of micturition and urgency [53]. Physiological circadian oscillation of detrusor muscle Cx43 participates in the diurnal variation in bladder capacity [21]. Multiple pathological factors have been identified to upregulate Cx43 expression such as inflammatory mediators, oxidative stress and growth factors in different cell types. Li et al. reported that proinflammatory cytokines potently increase Cx43 expression and function in bladder smooth muscle cells. Oxidative stress has been also shown to increase Cx43 in bladder smooth muscles [29]. Several growth factors including fibroblast growth factor (FGF) and NGF have been implicated in the increase of Cx43 level and function [49, 54]. Increased intercellular electrical coupling between adjacent detrusor cells allows easy spread of electrical activity and generate significant contractions, and involuntary intravesical pressure rises. When the condition is left untreated, irreversible changes in DSM occur with decreased bladder compliance, increased intravesical pressure during the bladder filling phase and dysfunction of the upper urinary tract [13]. Activation of A MPK has been shown to decrease Cx43 expression via the inhibition of CREB; a transcription factor that binds to cAMP response element sites and controls various cellular activities [21]. This may explain, at least in part, how the treatment with CoQ10 may have decreased CX43 expression levels in the current study. However, it does not point clearly whether this is a direct effect or secondarily to decreased inflammation and oxidative stress, or NGF level.

## Conclusion

The current study demonstrates that MSG can alter detrusor smooth muscle activity with possible detrimental effects on bladder storage and voiding functions. The inflammatory and oxidative stress consequences of ingesting MSG led to increase in urinary bladder tissue level of NGF and Cx43expression; resulting in increased sensitization and probably electrical coupling of detrusor muscles. The use of the multi-modal AMPK activator CoQ10 could be a potential adjuvant treatment. CoQ10 possesses anti-inflammatory and anti-oxidative properties, enabling it to play a possible therapeutic role.

## Data Availability

Data supporting findings are presented within the manuscript.

## Abbreviations

AMPK: Adenosine monophosphate-activated protein kinase
CAT: Catalase
CoQ10: Coenzyme Q10
DSM: Detrusor smooth muscle
FGF: Fibroblast growth factor
IL-6: Interleukin 6
MDA: Malondialdehyde
MSG: Monosodium glutamate
NGF: Nerve growth factor
SOD: Superoxide dismutase
STP: Sodium thiopental
TNF-α: Tumor necrosis factor alpha
